# Supplementary material for: Acupuncture for treating attention deficit hyperactivity disorder in children: A protocol for systematic review and meta-analysis
Source: PLoS One. 2022 Oct 10;17(10):e0275504. doi: 10.1371/journal.pone.0275504 (PMC9550064; doi:10.1371/journal.pone.0275504)
Supplement: Attachment — Submitted filename: Responses to reviewers comments.docx [file pone.0275504.s003.docx]

Dear Editor

On the behalf of my co-author, I would like to thank you for arranging peer-review of our manuscript and for your invitation to submit a revised version. We have highlighted the revised points according to valuable comments. We appreciate the effort of the reviewers and believe that their constructive suggestions have resulted in a stronger manuscript for the PLoS One’s readers.

Yours faithfully,

Myeong Soo Lee, PhD on the behalf of co author

**Additional Editor Comments:**

**Comment 1)** It is essential that you clarify how you will handle cases where studies do not comply to standards (lack of STRICTA protocol, lack of sufficient information, impossibility to contact the PI of the study, etc) and in which cases you will discard the study for lack of quality

**Revised>** Thank you very much for your valuable comments. We have now changed the original sentences as following sentences (**P. 7, Data extraction, 2^nd^ paragraph**).

*“In cases where studies do not meet standards (lack of STRICTA protocol, insufficient information), we will email or call the authors for additional information and note the results in the final publication. We will also report the case where it is not possible to contact the PI of the study and obtain the full information. We will include all eligible studies, regardless of missing information and poor quality, to obtain complete evidence.”*

**Reviewers' comments:**

**Comment 1)**: Page 3 - "Acupuncture is mainly widely used" - 'mainly widely' does not read well. Suggest rephase to "Acupuncture is widely used in Asia"

**Revised>** We have now changed it as commented (**P. 3, 2^nd^ paragraph, line 6**).

**Comment 2)** Page 3 - five lines from the bottom "one contained no eligible studies". I do not understand what the authors mean here. If no studies were eligible, does it mean that the review showed that there are zero studies that asses the use of acupuncture in children with ADHD? Eligible in what sense?

**Revised>** Thank you so much for your valuable comment. We have now clarified this as followings (**P.3, lines 4-5 from the bottoms**).

*“One of these three were Cochrane reviews, but they found no eligible studies [19].”*

**Comment 3)** Page 3 - last line "They concluded biased or exaggerated conclusions". This almost seems like a contradiction to the previous comment that these studies indicted limited evidence. If the evidence is limited and exaggerated, this implies that there is in fact no evidence. I would suggest "Further, the authors combined the studies regardless of control treatments and ignored clinical heterogeneities, resulting in potentially biased or inaccurate conclusions"

**Revised>** We have now changed it according to your valuable comments (**P 3, lines 1-2 from the bottoms**).

**Comment 4)** Page 4 - line 2. Rephase "...systematic review will aim to provide and up-to-date evaluation on the effects...."

**Revised>** We have now changed it according to your valuable comments (**P. 4, line 2**).

**Comment 5)** Page 4 - Under types of studies. Specify which languages will be used for the searches.

**Revised>** Thank you so much for your valuable comment. We have now added this under the Electric searches as followings (**P. 6, lines 5-6**).

*“We will use English for the search terms of all eight databases. In addition, we will use Korean for RISS and KISS and Chinese for CNKI.”*

**Comment 6)** Page 4 to 5 - In types of interventions and controls, how will studies that include alternative therapies alongside acupuncture (eg cupping, exercise) be handled?

**Revised>** Thank you so much for your valuable comment. We have now added this as followings (**P. 5, lines 8-9**).

*“We will exclude RCTs comparing two different forms of acupuncture or using other types of alternative therapies including cupping, herbal medicines, exercise and etc.”*

**Comment 7)** Page 5 lines 5 to 6, I find this sentence clumsy. Rephrase the sentence to clarify that combined treatments will be considered eg. "we will also include studies that compared the active treatment with acupuncture, as well as studies that combined the active treatment with acupuncture"

**Revised>** We have now changed it according to your suggestion (**P. 5, lines 6-8**).

**Comment 8)** Page 6 - "searching reference lists of the selected studies" is written 3 times on this page.

**Revised>** We have now corrected them according to your valuable comments (**P. 6, 2^nd^ paragraph, lines 5-7**).

**Comment 9)** Page 6 search strategy. Is it worth adding "ADHD" to the search?

**Revised>** We have now added it according to your valuable comments (**P. 6, Search strategies, line 3**).

**Comment 10)** Page 7 Assessment of risk bias. This paragraph has been written in the past tense. I think it should be written in future tense, assuming it is relevant to the proposed analyses.

**Revised>** We have now corrected them (**P. 7, lines 2-3 from the bottoms, P. 8, lines 1-2**).

“We will assess the risk of bias based on the Risk of Bias Assessment Tool (RoB 2.0) developed by the Cochrane Collaboration [28]. Five aspects will be examined: randomization, deviations from planned interventions, missing outcome data, measurement of outcomes, and selection of reported outcomes. Risk of bias will be graded as "low risk of bias," "some concern," or "high risk of bias" for each area of each study. Disagreements will be resolved by involving a third reviewer when necessary.”

**Comment 11)** Data: Given that the authors propose performing subgroup analyses, data from studies included in the review will need to be obtained / downloaded. These data should be made available and how this will be done should be discussed within the text.

4. Have the authors described where all data underlying the findings will be made available when the study is complete?

The PLOS Data policy requires authors to make all data underlying the findings described in their manuscript fully available without restriction, with rare exception, at the time of publication. The data should be provided as part of the manuscript or its supporting information, or deposited to a public repository. For example, in addition to summary statistics, the data points behind means, medians and variance measures should be available. If there are restrictions on publicly sharing data—e.g. participant privacy or use of data from a third party—those must be specified.

**Revised>** We have now added it as followings according to your valuable comments (**P. 9, lines 1-2. P.10. Data availability**).

***P.9, Lines 1-2***

*“We will provide all data underlying the results of this study in the article and supplementary materials.”*

***Data availability***

*“The authors confirm that the data supporting the findings of this study will be fully available within the article and its supplementary materials. Further inquiries can be directed to the corresponding author.”*
