# Supplementary material for: Acupuncture for treating attention deficit hyperactivity disorder in children: A protocol for systematic review and meta-analysis
Source: PLoS One. 2022 Oct 10;17(10):e0275504. doi: 10.1371/journal.pone.0275504 (PMC9550064; doi:10.1371/journal.pone.0275504)
Supplement: Attachment — Submitted filename: Responses to reviewers comments-PONE-D-22-17595R3.docx [file pone.0275504.s004.docx]

Dear Editor

On the behalf of my co-author, I would like to thank you for arranging peer-review of our manuscript and for your invitation to submit a revised version. We have highlighted the revised points according to valuable comments. We appreciate the effort of the reviewers and believe that their constructive suggestions have resulted in a stronger manuscript for the PLoS One’s readers.

Yours faithfully,

Myeong Soo Lee, PhD on the behalf of co author

**Reviewers' comments:**

**Comment 1)** Thank you for taking the time to make the proposed changes. I am satisfied that all previous comments have been considered. I have only one remaining concern regarding the following comment:

"We will include all eligible studies, regardless of missing information and poor quality, to obtain complete evidence."

I do not agree that poor quality studies should be included as this may lead to misuse and poor-quality results further down the line. If the authors wish to include these studies in their report, then I think that it should be explicitly stated that "whilst all studies that meet the search criteria will be considered, those that do not meet the proposed standards will be listed separately with the reason for exclusion noted". Reasons for exclusion should then be listed in a manner that it not ambiguous, eg "insufficient information" should state what was missing and how much was missing. Only those studies that meet the inclusion criteria and none of the exclusion criteria should be presented in the results tables.

**Revised>** We have now changed it as commented (**P. 7, lines 2-5 from the bottoms**).

*“While all studies that meet the search criteria will be included, those that do not meet the proposed standards will be listed separately and the reason for exclusion is noted. Only those studies that meet the inclusion criteria and none of the exclusion criteria will be included in the results tables.”*
